# Letter from R. C. Gercke

**Published:** 1891-02

**Authors:** R. C. Gercke

**Affiliations:** Augusta, Ga.


*Editors Atlanta Medical and Surgical Journal:*

GENTLEMEN—I have lately read accounts of accidents to persons, whereby the whole head or other parts of the body were deprived of their natural covering, which cases, when put under the care of physicians, proved a source of worry and trouble, by reason of the doctor's inability of procuring necessary grafts for re-covering the denuded parts, very few people being found willing to furnish the required (parts) grafts.

Some time ago, I advised a physician in such a case. The skin of frogs had been tried without much benefit. As others may be troubled in the same way, I give, for their benefit, the advice I gave.

I advised the doctor to visit a hospital, and pick out from the cases *in articulo mortis* one as much like his patient, in color of hair, age, etc., as possible, and ask the attending physician to supply him with the necessary amount of grafts. These can easily be furnished when the party is unconscious before death, or even after life has departed.

I speak from experience, as during several years past I have used such grafts myself.

Even after a patient's death, parts of skin transplanted to a living patient's body answered the purpose quite well.

I am convinced that any part, to form skin or bone, or whatever is needed, could be used from persons just deceased, provided it remained in a healthy condition, and was not affected by the last sickness. However, there would probably be more failures with grafts taken after than with such as were taken before death. Very truly yours,

R. C. GERCKE.

Augusta, Ga., January 28th, 1891.

